# Examine the Impact of Self‐Medicated Antibiotics on Gut Bacterial Diversity From COVID‐19 Patients in Gopalganj, Bangladesh

**DOI:** 10.1002/mbo3.70063

**Published:** 2025-09-30

**Authors:** Rabeya Khanam, Md. Yamun Hasan, Abdul Malek, Sazzad Hossain Sagor, Chandan Sikder, Md. Sahabuddin

**Affiliations:** ^1^ Department of Biotechnology and Genetic Engineering Gopalganj Science and Technology University Gopalganj Bangladesh; ^2^ Biotechnology and Genetic Engineering Discipline Khulna University Khulna Bangladesh; ^3^ Department of Microbiology and Hygiene, Faculty of Veterinary Science Bangladesh Agricultural University Mymensingh Bangladesh

**Keywords:** antimicrobial resistance, COVID‐19, Gram‐negative bacteria, gut microbes, multidrug resistant, self‐medication

## Abstract

The COVID‐19 pandemic has claimed millions of lives globally and continues to pose a threat to public health. It has influenced the self‐medication (SM) of antibiotics during the pandemic due to coronaphobia, similar symptoms to flu, cold, respiratory illnesses, insufficiency of reverse transcription polymerase chain reaction (PCR) test, and easy access to the drug, and so forth. Self‐medication with antibiotics (SMA) raises the resistance profile of gut bacteria to antibiotics. This cross‐sectional study evaluated the connection between antibiotic consumption and the antibiotic‐resistant patterns of gut bacteria isolated from 29 COVID‐19 patients in Gopalganj, Bangladesh. Standard microbiological tests and molecular methods such as PCR and *16S rRNA* sequencing were performed for bacterial identification. The disk diffusion method was used for antibiotic susceptibility testing. A total of 48 bacterial isolates, including *Escherichia coli* (*n* = 24), *Klebsiella pneumoniae* (*n* = 7), *Pseudomonas aeruginosa* (*n* = 7), *Salmonella* spp. (*n* = 4), *Shigella flexneri* (*n* = 3), *Providencia alcalifaciens* (*n* = 1), *Proteus vulgaris* (*n* = 1), and *Yersinia enterocolitica* (*n* = 1), were identified. The prevalence of SM and multidrug resistance patterns was 65.5% and 89.58%, respectively. Self‐medicated COVID‐19 patients reported higher antibiotic resistance than patients who consumed prescribed antibiotics regularly. This study demonstrated that, in addition to SMA, other factors such as diet, water, sanitation, and so on can contribute to the development of antibiotic‐resistant bacteria. A healthy lifestyle and awareness while using antibiotics can limit our gut microbes from becoming antimicrobial‐resistant.

## Graphical abstract

Our research found that people in Bangladesh had a considerable increase in antibiotic resistance in their gut bacteria due to self‐medication with antibiotics during the COVID‐19 pandemic. To prevent the spread of antibiotic‐resistant bacteria, our research highlighted the crucial role of raising awareness and using antibiotics appropriately.

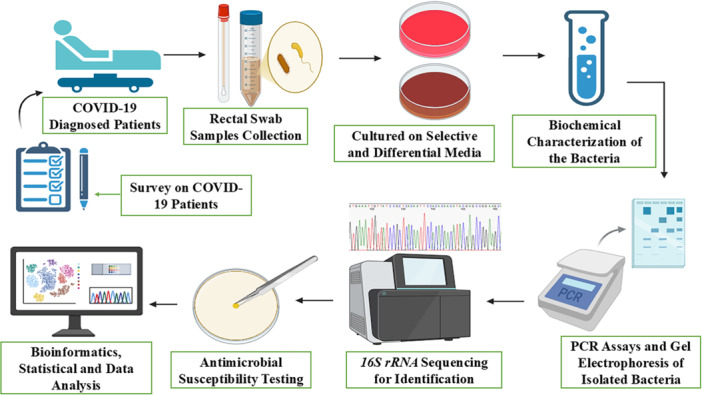

## 1 Introduction

COVID‐19 is a viral infection caused by the “Severe Acute Respiratory Syndrome Coronavirus 2 (SARS‐CoV‐2),” which mostly infects the respiratory tract (Zuo et al. 2021). In March 2020, the World Health Organization (WHO) officially declared COVID‐19 a worldwide pandemic. Since its emergence, SARS‐CoV‐2 has become a global concern due to its rapid transmission across countries and its ability to cause a wide range of clinical illnesses, from mild to severe inflammatory conditions that can lead to multiorgan failure and mortality (Abdelmalek and Mousa 2022). The COVID‐19 pandemic has significantly impacted Bangladesh, with a total of 2,049,377 confirmed cases and 29,493 fatalities (Worldometer 2024). The COVID‐19 pandemic has significantly impacted developing countries because of the limited availability of healthcare services, and this condition has increased people's self‐medication (SM) with medicines (Khoshbakht et al. 2023). WHO defined SM as the act of an individual treating their illness symptoms with medications without a prescription or consulting a physician (Saha et al. 2022; Subashini and Udayanga 2020). During the COVID‐19 pandemic, concerns about contracting the virus, the absence of a proven treatment plan, the initial lack of vaccines, the increase of misleading information, and difficulties in accessing medical facilities due to lockdowns and restrictions have all increased SM (Khami et al. 2022; Zheng et al. 2023). Research conducted in Iran with COVID‐19 outpatients found that 59.6% of them took self‐treatment (Faraji et al. 2022). On the basis of a comprehensive analysis conducted across various continents of Arab regions, it was found that a significant proportion of the participants, approximately 62.7% took SM (Abdelwahed et al. 2023). In addition, a recent systematic review found that antibiotics were the most commonly utilized self‐medicated medication during COVID‐19 (79%) (Khoshbakht et al. 2023).

Although COVID‐19 is a viral disease, using antibiotics can help ameliorate further bacterial coinfections associated with viral respiratory infection (Rizvi and Ahammad 2022). No clinical evidence was found about the public misinformation regarding the use of antibiotics to treat COVID‐19. Although physicians prescribed antibiotics to prevent coinfections at the time of the pandemic (Abdelmalek and Mousa 2022). A study conducted by Langford et al. (2021) revealed that over 75% of patients were given antibiotics, despite a relatively low incidence of coinfection. Self‐medication with antibiotics (SMA) has recently become an important threat to public health in underdeveloped and developing countries (Xavier et al. 2022). A recent study on Indigenous people in Bangladesh found that 48.7% used antibiotics by SM (Mannan et al. 2024). A cross‐sectional survey involving 1000 Bangladeshi students revealed that approximately 61% of the participants experienced SMA, which indicates that SM practices among educated people increased alarmingly (Wahab et al. 2023). The improper use of antibiotics by SM has contributed to antibiotic resistance, toxicity of drugs, antibiotic‐associated diarrhea, disruption of the gut microbiome, and so forth (Morgan et al. 2011). A metagenomics study demonstrated that the gut microbial composition in COVID‐19 patients displayed a significant change with a decrease in beneficial microbes and an increase in opportunistic pathogenic microbes compared with healthy people (Sun et al. 2022). Moreover, a notable rise in the prevalence of common opportunistic pathogens, specifically Enterobacteriaceae (*Klebsiella* spp., *Salmonella* spp., *Shigella* spp., and enterotoxigenic *Escherichia coli*), *Enterococcus* (*Streptococcus* and *Ruminococcus*), and *Pseudomonas* spp., among patients with COVID‐19 increases pro‐inflammatory cytokines, which leads to cytokine storms and ultimately contributes to disease severity (van der Lelie and Taghavi 2020; Tang et al. 2020). Gut bacterial species contribute significantly to gastrointestinal equilibrium by regulating the host's immunological responses (Hussain et al. 2021). Changes in gut microbiota can contribute to a hyperactive immune system, which increases the risk of respiratory disorders like asthma, allergic reactions in the lungs, and chronic respiratory diseases (Enaud et al. 2020; Marsland et al. 2015).

Overuse and misuse of antibiotics lead to the development of antimicrobial resistance (AMR) and contribute to multidrug‐resistant (MDR) strains of bacteria. In the 21st century, AMR is a significant health burden that results from bacterial changes and the development of resistance to antibiotics (Xavier et al. 2022). In 2019, the WHO reported that antibiotic resistance resulted in 1.27 million global deaths and contributed to 4.95 million deaths globally by AMR, overtaking the number of deaths due to HIV/AIDS or malaria, with a significant impact on low‐ and middle‐income countries (LMICs) (Murray et al. 2022). According to the Institute for Health Metrics and Evaluation (IHME), 26,200 deaths were caused by AMR in 2019 and a total of 98,800 deaths occurred due to AMR in Bangladesh (IHME 2023). The gut bacteria act as a reservoir for antimicrobial resistance genes (ARGs), which possibly occur by horizontal gene transfer from other strains or misuse of antibiotics (Mahmud et al. 2020; Su et al. 2022). A different study with 120 samples collected from chicken meats and eggs, where 50% were identified as *E. coli*, 17% as *Salmonella* spp., and 32% as *S. aureus*, and most isolates were MDR (Rafiq et al. 2024). A study suggested that in Bangladesh, street foods contain AMR *E. coli* isolates, which may contribute to the spreading of resistance genes to other commensal bacteria (Hasan et al. 2021). These ARGs may easily spread to commensal and opportunistic bacteria, resulting in a global problem of drug‐resistant species that limit treatment choices (Su et al. 2022).

Gut bacteria with MDR pose a global health issue because of the easy transmissibility of AMR to commensal strains, which increases treatment costs, hospital stays, and serious infections. Most of the research conducted previously on the impact of SM of antibiotics during COVID‐19 was limited to survey‐based studies (Abdelwahed et al. 2023; Faraji et al. 2022; Khami et al. 2022; Mannan et al. 2024; Rizvi and Ahammad 2022), meta‐analysis (Zuo et al. 2021), and reviews (Langford et al. 2021; Zheng et al. 2023). However, very few studies were done, but no study has given the actual scenario about the impact of self‐medicated antibiotics on gut bacteria, in particular, no research has been conducted yet in Bangladesh focusing on antibiotic‐resistant patterns of patients with COVID‐19 using antibiotics. Therefore, this study aimed to evaluate the resistance patterns in the gut bacteria of COVID‐19 patients and investigate the relationship between antibiotic resistance patterns and other associated factors like antibiotic consumption timeline, maintaining sanitation, processing poultry meats and eggs before consumption, drinking water, residents, and pet animals.

## 2 Materials and Methods

### 2.1 Study Area, Sampling, and Sample Processing

In this study, samples along with a structured survey form were collected from several locations in Gopalganj District, Bangladesh, between June 2022 and December 2023. Using sterile cotton swab sticks and phosphate buffer solution, 29 rectal swab samples were collected from COVID‐19 patients. Samples were immediately transferred to the Biotechnology and Genetic Engineering Laboratory of Gopalganj Science and Technology University, Goalaganj‐8100, Bangladesh, and stored at 4°C until further analysis. Samples were frequently diluted in the lab by a factor of 10^−5^ for further experiments.

### 2.2 Isolation of the Bacterial Isolates

Each diluted sample was cultured in MacConkey agar (Hi‐Media, India) and eosin methylene blue agar (Hi‐Media, India) by the spread‐plating technique and incubated for 24 h at 37°C. To obtain the pure colonies, each isolate's cultural characteristics, such as shape, size, and color of each colony, were observed and transferred to appropriate selective agar and then incubated overnight at 37°C. Suitable agar media were used to store the pure colonies of each isolated bacterium at 4°C for biochemical characterization and molecular analysis.

### 2.3 Biochemical Characterization of the Isolates

Biochemical characterization of each isolate was done using morphological characteristics such as Gram staining properties and several other tests, such as lactose fermentation, oxidase, catalase, methyl red (MR), Voges–Proskauer, motility, indole, and urease, according to standard bacteriological protocols (Farmer et al. 1985; Humphries and Linscott 2015; Weyant 1996). For validation, each biochemical test was performed 2–3 times.

### 2.4 DNA Extraction

Bacterial DNA was extracted using the Wizard Genomic DNA Purification Kit (Promega, USA) following the guidelines provided by the manufacturer. The extracted DNA was stored at a temperature range of 2°C–8°C. DNA quantification was carried out by using a spectrophotometer, NanoDrop 2000c (Thermo Fisher Scientific, USA).

### 2.5 Polymerase Chain Reaction (PCR) Assays and Gel Electrophoresis

*16S rRNA gene* of the extracted DNA was amplified using universal primers 27 F (5′‐AGAGTTTGATCCTGGCTCAG‐3′) and 1492 R (5′‐TACGGYTACCTTGTTACGACTT‐3′) (Dos Santos et al. 2019; Heuer et al. 1997). PCR assays were carried out in a final volume of 25 μL reaction mixture that contained GoTaq G2 Hot Start Master Mix 12.5 μL (Promega, USA), Template DNA 1 μL (concentration 25–65 ng/μL), Primer Forward and Reverse 1 μL each (concentration 10–20 pMol), and nuclease‐free water 9.5 μL. The conditions for the thermocycling of the PCR amplification were as follows: preheat at 95°C for 3 min, following 35 cycles of denaturation at 95°C for 30 s, annealing at 49°C for 30 s, and strand extension at 72°C for 90 s; a final extension at 72°C for 5 min; and finally, the PCR products held for overnight at 4°C temperature.

After that, PCR products were analyzed using gel electrophoresis at 90 V for 30 min in a 1% agarose gel (Hi‐Media, India) with ethidium bromide as a stain. A DNA ladder with a length of 1000 bp (Promega, USA) was used to determine the size of the target amplicon of the amplified gene. Results were visualized under an ultraviolet transilluminator called AlphaImager Mini (ProteinSimple, USA).

### 2.6 Purification and *16S rRNA Gene* Sequencing of the PCR Products

The PCR products were purified following the standard protocols from the Wizard SV Gel and PCR Clean‐Up System (Promega, USA). The purified eluted DNA was stored at −20°C. Bacteria were identified using *16S rRNA gene* sequencing. In cases of closely related species, especially *E. coli* and *Shigella* spp., several biochemical tests (such as lactose fermentation, indole, and motility) were performed, along with *16S rRNA gene* sequencing, as a secondary identification method. The PCR products were sequenced using Sanger's sequencing technique on an Applied Biosystems 3500 series genetic analyzer (Thermo Fisher Scientific, USA) at Apical Scientific Laboratory in Selangor, Malaysia. The initially generated sequence of each primer was trimmed to remove any potentially misleading data from the sequencing fragments. The sequences were assembled into a consensus sequence using Bio‐Edit software (V7.2).

### 2.7 Antimicrobial Susceptibility Testing

The antimicrobial susceptibility of the isolated bacteria was tested against commonly used antibiotics with the Kirby–Bauer disc diffusion method in Muller Hinton agar (Hi‐Media, India) following protocols of the Clinical and Laboratory Standards Institute (CLSI) (Lewis II et al. 2024). Antibiotic discs (Bioanalyze, Turkey) used in the study included penicillins (penicillin G, 10 μg; ampicillin, 10 μg; amoxicillin, 10 μg; piperacillin, 100 μg), macrolides (azithromycin, 15 μg; erythromycin, 15 μg), tetracyclines (tetracycline, 30 µg; doxycycline, 30 μg), cephalosporins (cefixime, 10 µg; cefuroxime, 30 µg; ceftriaxone, 30 µg), phenicols (chloramphenicol, 30 µg), and aminoglycosides (gentamicin, 10 µg). The measurement of growth inhibition zones was observed following incubation at 37°C for 16–18 h. The isolates were categorized into resistant, intermediate, and susceptible groups by measuring the size of the inhibitory zone. As control standard strains for antimicrobial susceptibility testing, *E. coli* strain ATCC 25922 and *Pseudomonas aeruginosa* strain ATCC 27853 were used. MDR strains were identified based on their nonsusceptibility to at least three different classes of antibiotics (Magiorakos et al. 2012).

### 2.8 Bioinformatics and Statistical Analysis

The assembled sequences were cross‐referenced with the National Center for Biotechnology Information (NCBI) databases. The nucleotide blast algorithms and the Basic Local Alignment Search Tool from the NCBI (https://blast.ncbi.nlm.nih.gov/Blast.cgi) were used for assessing the degree of similarity with other sequences. The *16S rRNA gene* sequence was used to identify isolates at the species level when there was a > 99% identity and at the genus level when there was a 97%–99% identity (Drancourt et al. 2004). Sequences generated in this study were submitted to GenBank. After that, these sequences, along with other relevant sequences from GenBank, were aligned using MAFFT (V7.526) to construct a phylogenetic neighbor‐joining tree using The Interactive Tree of Life (iTOL V6) (Letunic and Bork 2024).

The data was evaluated using SPSS software (16th edition). Descriptive statistical tests were conducted as follows: categorical variables were examined using frequency and percentages, while continuous variables were analyzed using means and standard deviations (SDs). The graph was constructed using the following legacy dialogs in the bar chart. A Chi‐square test was used to analyze statistically significant correlations (*p* < 0.05) among antibiotic resistance levels (low, medium, and high) with antibiotic consumption timelines, consuming street foods, maintaining sanitation, processing poultry and eggs, drinking water, residents, and pet animals.

## 3 Results

### 3.1 Characteristics of COVID‐19 Patients

A total of 29 patients from different time frames of the COVID‐19 wave went through clinical microbiological testing, among them 89.7% were male (*n* = 26), and 10.3% were female (*n* = 3). The age of the patients was between 20 and 34 years with a mean age of (22.97 ± 2.542). The records of antibiotics used by patients during COVID‐19 (isolation time), post‐COVID‐19 (within 6 months before sample collection), pre‐COVID‐19 (before 2 years of COVID‐19) by SM or doctor‐prescribed and their lifestyle including consuming street foods, having pet animals, residents, source of drinking water, sanitation, and involvements of food processing (poultry meats and eggs) are outlined in Table 1.

**Table 1:** COVID‐19 patient characteristics based on survey data.

Criteria	Frequency
Gender of patients	Male, *n* = 26 (89.7)
(*n*%)	Female, *n* = 3 (10.3)
Antibiotics taken during COVID‐19 (isolation time)	Yes, *n* = 19 (65.5%)
(*n*%)	No, *n* = 10 (34.5%)
Antibiotics taken post‐COVID‐19 (within 6 months before sample collection)	Yes, *n* = 6 (20.7)
(*n*%)	No, *n* = 23 (79.3)
Antibiotics taken pre‐COVID‐19 (last 2 years before COVID‐19)	Yes, *n* = 25 (86.2)
(*n*%)	No, *n* = 4 (13.5)
Consumption of antibiotics in three timelines (during COVID‐19, post‐COVID‐19, and pre‐COVID‐19)	Yes, *n* = 25 (86.2)
(*n*%)	No, *n* = 4 (13.8)
Self‐medication (SM)	Yes, *n* = 19 (65.5%)
(*n*%)	No, *n* = 10 (34.5%)
Consumption of street food in a week	Every day, *n* = 12 (41.4)
(*n*%)	Once a week, *n* = 7 (24.1)
	Twice a week, *n* = 10 (34.5)
Having pet animals	Yes, *n* = 5 (17.2)
(*n*%)	No, *n* = 24 (82.8)
Residents	Local mess, *n* = 24 (82.8)
(*n*%)	University hall, *n* = 5 (17.2)
Source of drinking water	Locally supplied water, *n* = 24 (82.8)
(*n*%)	University hall, *n* = 5 (17.2)
Maintained sanitation	Yes, *n* = 11 (37.9)
(*n*%)	No, *n* = 18 (62.1)
Processing poultry meats or eggs before consumption	Yes, *n* = 16 (55.2)
(*n*%)	No, *n* = 13 (44.8)

### 3.2 Prevalence of Bacterial Isolates

**Figure 1:**
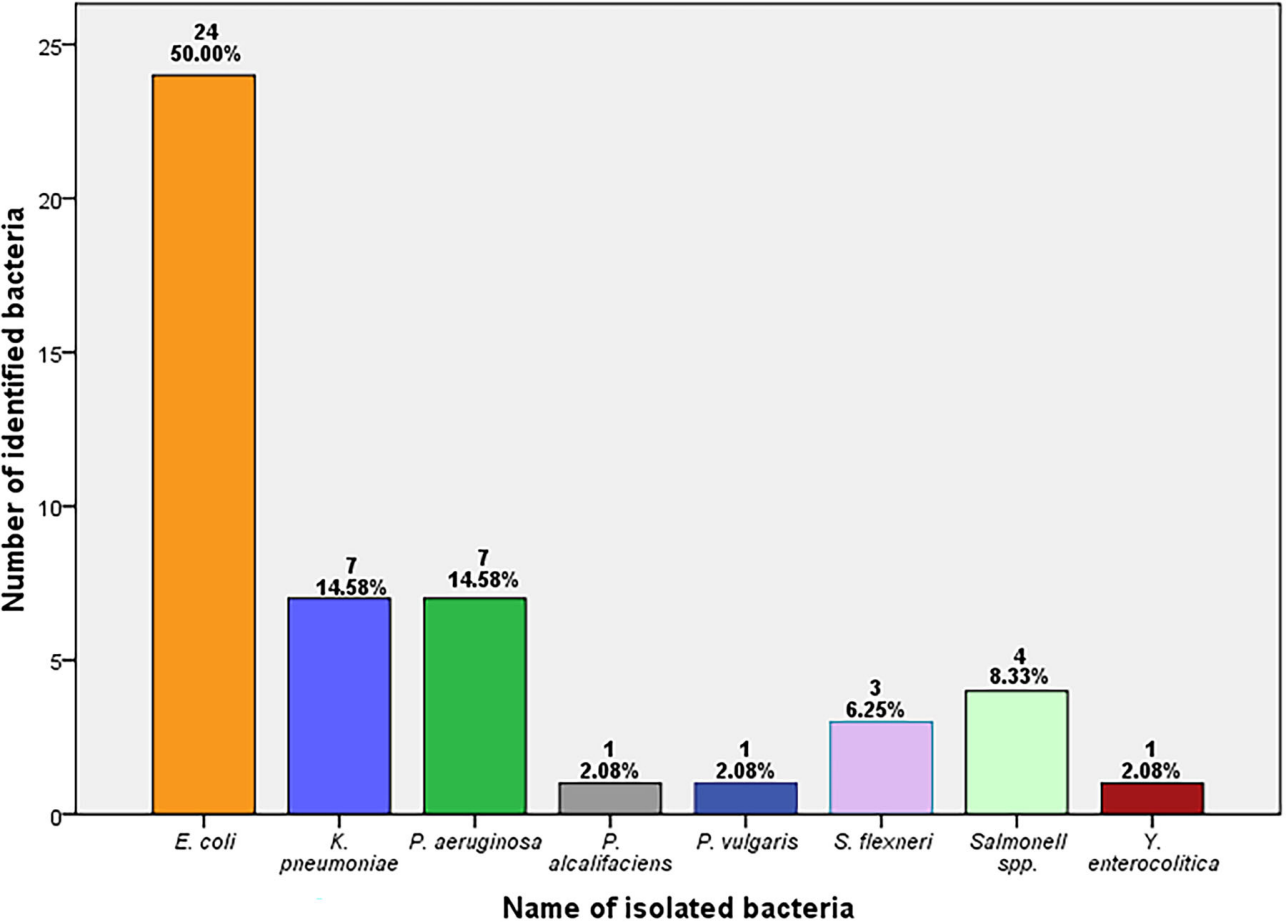
Number of bacteria isolated from COVID‐19 patients. *Escherichia coli*, *Klebsiella pneumoniae*, *Proteus vulgaris*, *Providencia alcalifaciens*, *Pseudomonas aeruginosa*, *Shigella flexneri*, and *Yersinia enterocolitica*.

A total of 48 Gram‐negative bacterial isolates were identified from 29 specimens using standard biochemical tests, which are provided in Table S1. Among the isolates, surprisingly half of them were *E. coli* (*n* = 24, 50%), and an equal number of isolates were obtained between *Klebsiella pneumoniae* (*n* = 7, 14.58%) and *P. aeruginosa* (*n* = 7, 14.58%), which were followed by *Providencia alcalifaciens* (*n* = 1, 2.08%), *Proteus vulgaris* (*n* = 1, 2.08%), and *Yersinia enterocolitica* (*n* = 1, 2.08%). The obtained number of *Shigella flexneri* (*n* = 3, 6.25%) and *Salmonella* spp. (*n* = 4, 8.33%) were quite similar, and all the obtained Gram‐negative bacteria are listed in Figure 1.

### 3.3 PCR Assays and Gel Electrophoresis

**Figure 2:**
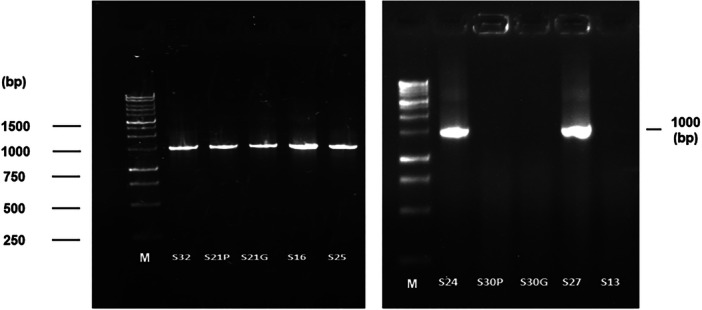
Gel electrophoresis analysis of *16S rRNA gene* generated from 10 bacterial isolates.

Universal Primer 27 F and 1492 R were used to amplify the extracted bacterial DNA of 10 isolates. The first lane in both gels was used as a marker, denoted as M (1 kb DNA ladder). The intense and sharp band was observed in seven isolates (S‐32, S‐21P, S‐21G, S‐25, S‐24, and S‐27) among the 10 isolates in gel electrophoresis, and their size was approximately 1000 bp. The results are presented in Figure 2. Three isolates (S‐30P, S‐30G, and S‐13) did not exhibit any band in the gel because of the lack of nucleic acid per unit (ng/μL).

### 3.4 Molecular Identification of Isolates by *16S rRNA Gene* Sequencing and Phylogenetic Analysis

Among 48 isolates, eight types of Gram‐negative bacterial isolates (*E. coli*, *K. pneumoniae*, *P. aeruginosa*, *S. flexneri, Salmonella* Typhi, *P. alcalifaciens, P. vulgaris*, and *Y. enterocolitica)* were identified using *16S rRNA gene* sequencing were as follows: MSN/BD/S16, MSN/BD/S24, MSN/BD/S32 of *E. coli* (*n* = 3), MSN/BD/S21P of *P. aeruginosa* (*n* = 1), MSN/BD/S17, MSN/BD/S25 of *K. pneumoniae* (*n* = 2), MSN/BD/S25, and MSN/BD/S27 of *Salmonella enterica*. Table 2 presents the accession numbers obtained after submitting the sequences to NCBI.

**Table 2:** Isolated bacteria with an accession number from National Center for Biotechnology Information (NCBI).

S. No.	Strain type	Strain abbreviation	NCBI accession no.
1	*Escherichia coli*	MSN/BD/S16	PP999312
2	*E. coli*	MSN/BD/S24	PP999302
3	*E. coli*	MSN/BD/S32	PP999159
4	*Pseudomonas aeruginosa*	MSN/BD/S21P	PP999472
5	*Klebsiella pneumoniae*	MSN/BD/S17	PP999444
6	*K. pneumoniae*	MSN/BD/S25	PP999436
7	*Salmonella enterica*	MSN/BD/S25	PQ000984
8	*S. enterica*	MSN/BD/S27	PQ000246

**Figure 3:**
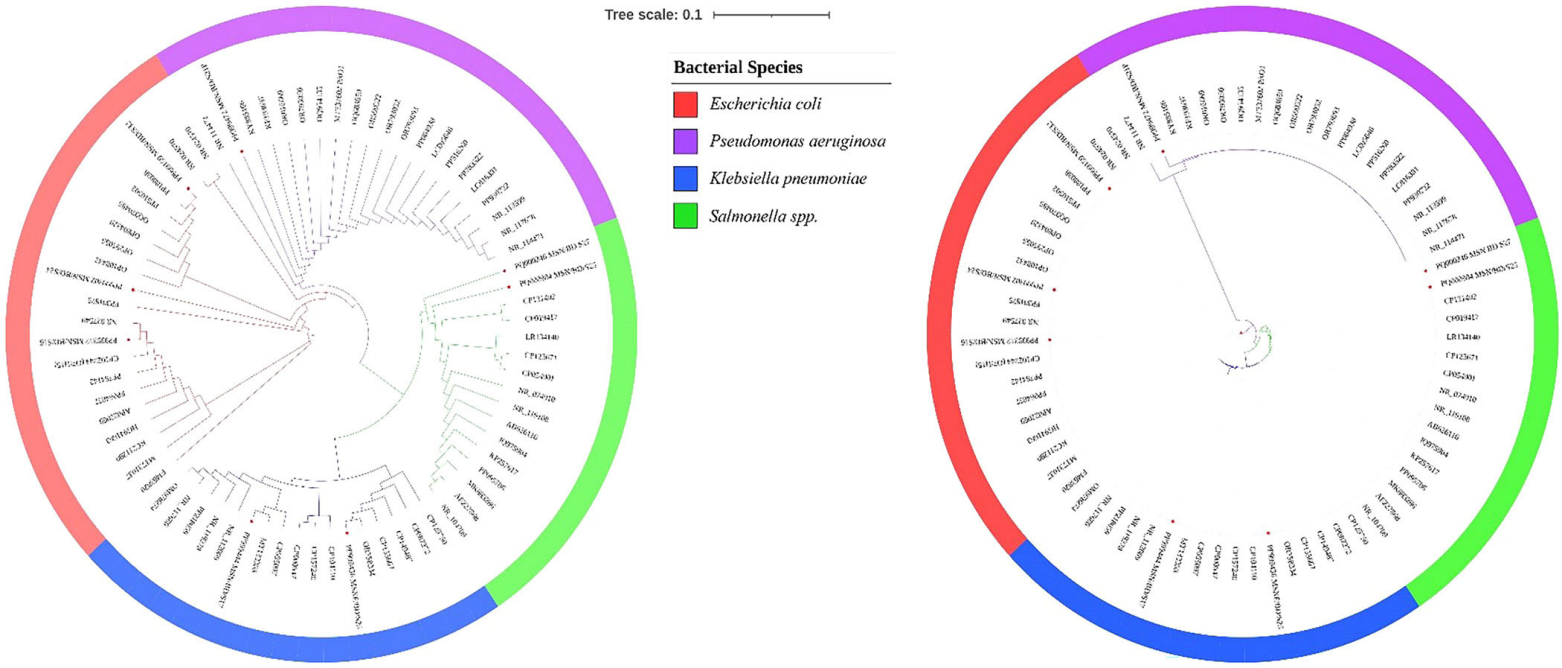
Phylogenetic tree constructed from the alignment of *16S rRNA gene* sequencing data. Strains were colored after the closest match to the strains retrieved from the GeneBank databases. The red color star marks indicate the gene sequence of this study.

A total of (*n* = 75) sequences were used to construct a phylogenetic tree by assembling the obtained sequences (*n* = 8) of this study and related retrieved sequences (*n* = 67) from NCBI for Phylogenetic analysis, which were shown in Figure 3.

### 3.5 Antibiotic Resistance Pattern of Isolated Bacteria From COVID‐19 Patients

#### 3.5.1 Antibiotic Resistance Pattern of COVID‐19 Patients Based on Their Antibiotic Consumption Practices

**Figure 4:**
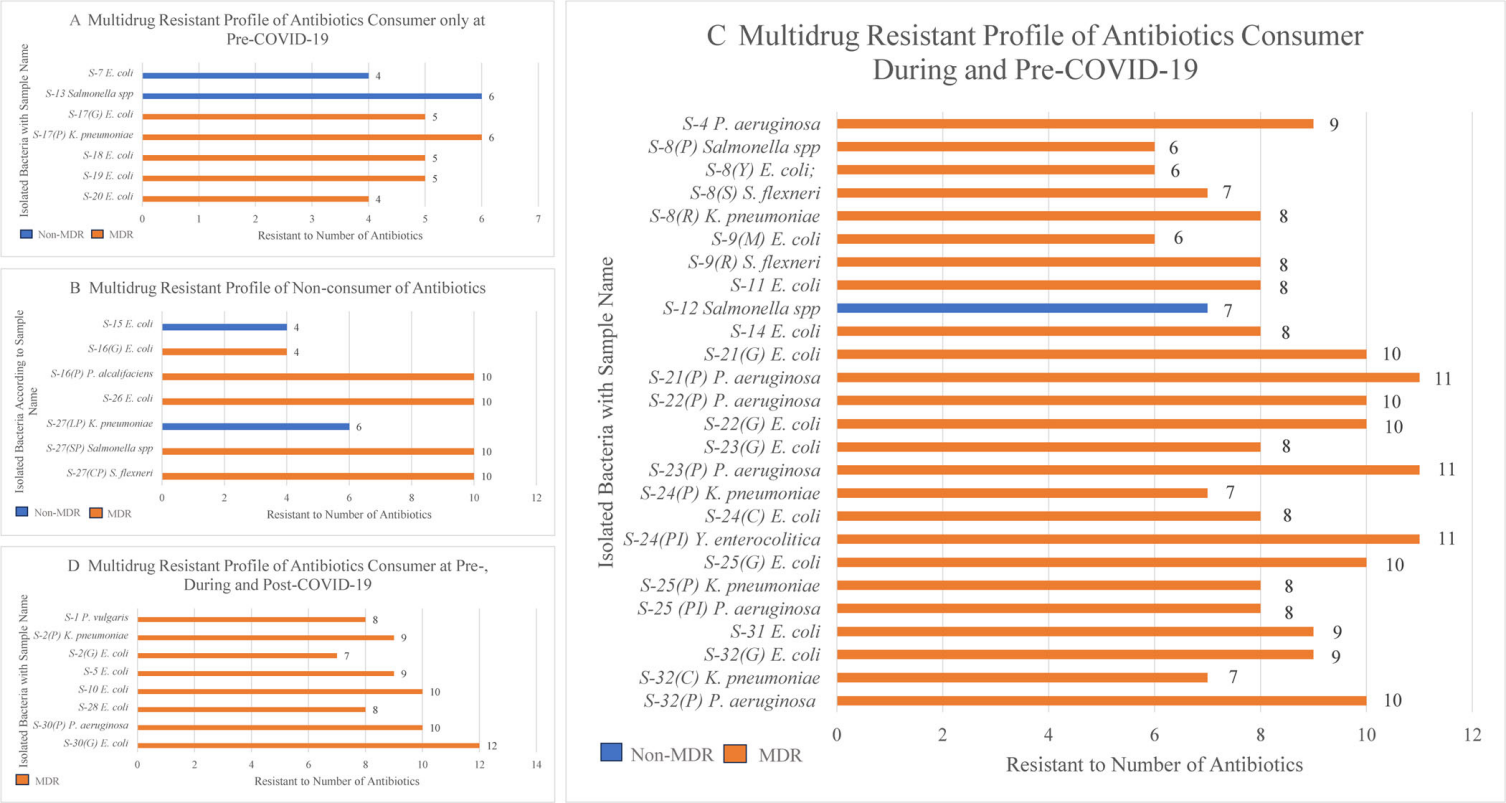
The relationship between the multidrug resistant (MDR) profile of bacterial isolates and the antibiotic consumption history of COVID‐19 patients. *Escherichia coli*, *Klebsiella pneumoniae*, *Proteus vulgaris*, *Providencia alcalifaciens*, *Pseudomonas aeruginosa*, *Shigella flexneri*, and *Yersinia enterocolitica*.

The COVID‐19 patient's antibiotic consumer groups and their isolated bacteria are summarized in Table 3. Depending on pre‐, during, and post‐COVID‐19, patients were divided into four groups, and their MDR profiles are shown in Figure 4A–D.

**Table 3:** Isolated bacteria from COVID‐19 patients according to their antibiotic consumption history.

Antibiotic consumer group	Sample numbers	Isolated bacteria
Nonconsumer of antibiotics (pre‐, during, and post‐COVID‐19), *n* = 4	S‐15, S‐16, S‐26, S‐27	S‐15 *Escherichia coli*; S‐16 (G) *E. coli*; S‐16 (P) *Providencia alcalifaciens*; S‐26 *E. coli*; S‐27 (LP) *Klebsiella pneumoniae*; S‐27 (SP) *Salmonella* spp.; S‐27 (CP) *Shigella flexneri*
Antibiotic consumed only pre‐COVID‐19, *n* = 6	S‐7, S‐13, S‐17, S‐18, S‐19, S‐20	S‐7 *E. coli;* S‐13 *Salmonella* spp.*;* S‐17 (G) *E. coli;* S‐17 (P) *K. pneumoniae;* S‐18 *E. coli;* S‐19 *E. coli;* S‐20 *E. coli*
Antibiotics consumed during and pre‐COVID‐19, *n* = 13	S‐4, S‐8, S‐9, S‐11, S‐12, S‐14, S‐21, S‐22, S‐23, S‐24, S‐25, S‐31, S‐32	S‐4 *Pseudomonas aeruginosa*; S‐8 (P) *Salmonella* spp.; S‐8 (Y) *E. coli;* S‐8 (S) *S. flexneri*; S‐8 (R) *K. pneumoniae*; S‐9 (M) *E. coli*; S‐9 (R) *S. flexneri*; S‐11 *E. coli*; S‐12 *Salmonella* spp.; S‐14 *E. coli*; S‐21 (G) *E. coli;* S‐21 (P) *P. aeruginosa*; S‐22 (P) *P. aeruginosa*; S‐22 (G) *E. coli*; S‐23 (G) *E. coli*; S‐23 (P) *P. aeruginosa*; S‐24 (P) *K. pneumoniae*; S‐24 (C) *E. coli*; S‐24 (PI) *Yersinia enterocolitica*; S‐25 (G) *E. coli*; S‐25 (P) *K. pneumoniae*; S‐25 (PI) *P. aeruginosa*; S‐31 *E. coli*; S‐32 (G) *E. coli*; S‐32 (C) *K. pneumoniae*; S‐32 (P) *P. aeruginosa*
Antibiotics consumed pre‐, during, post‐COVID‐19, *n* = 6	S‐1, S‐2, S‐5, S‐10, S‐28, S‐30	S‐1 *Proteus vulgaris*; S‐2 (P) *K. pneumoniae*; S‐2 (G) *E. coli*; S‐5 *E. coli*; S‐10 *E. coli*; S‐28 *E. coli*; S‐30 (P) *P. aeruginosa*; S‐30 (G) *E. coli*.

#### 3.5.2 Antibiotic Resistance Pattern of COVID‐19 Patients Based on Bacterial Isolates

In all, 13 antibiotics of six different classes were used in this study to evaluate the resistance pattern of the isolates (*n* = 48) from COVID‐19 patients presented in Tables 4 and 5. *E. coli* (*n* = 24) showed maximum resistance to penicillin (100%) which was mostly isolated during the study was followed by erythromycin (83.33%), amoxicillin (79.17%), cefixime (79.17%), piperacillin (70.83%), cefuroxime (70.83%), ampicillin (66.67%), azithromycin (62.5%), tetracycline (41.67%), gentamicin (41.67%), ceftriaxone (20.83%), chloramphenicol (20.83%), and doxycycline (16.67%). Among *K. pneumoniae* (*n* = 7), 100% were resistant against amoxicillin, azithromycin, ampicillin, and penicillin, and 100% of isolates exhibited susceptibility to ceftriaxone, gentamicin, and chloramphenicol. This research study also showed that *P. aeruginosa* (*n* = 7), *P. vulgaris* (*n* = 1), *P. alcalifaciens* (*n* = 1), and *Y. enterocolitica* (*n* = 1) all isolates were resistant to amoxicillin, azithromycin, ampicillin, penicillin, erythromycin, and cefuroxime. Among *S. flexneri* (*n* = 3), 100% of isolates were resistant against amoxicillin, azithromycin, ampicillin, penicillin, erythromycin, and cefixime, and 100% were susceptible to piperacillin, ceftriaxone, gentamicin, and chloramphenicol. All the *Salmonella* spp. (*n* = 4) isolates were resistant to amoxicillin and erythromycin and susceptible to chloramphenicol. They were also found resistant to azithromycin (50.0%), ampicillin (75.0%), erythromycin (50.0%), tetracycline (25.0%), cefixime (75.0%), piperacillin (75.0%), ceftriaxone (50.0%), gentamicin (25.0%), doxycycline (25.0%), and cefuroxime (75.0%).

**Table 4:** Antibiotic resistance profiling of identified Gram‐negative bacteria isolated from those specimens.

Identified bacteria	AMX	AZM	AMP	PEN	ERY	TET	CFX	PIP	CTR	GN	C		DOX	CRX
*Escherichia coli*, *n* = 24; *n* (%)	19 (79.17)	15 (62.5)	16 (66.67)	24 (100.0)	20 (83.33)	10 (41.67)	19 (79.17)	17 (70.83)	5 (20.83)	10 (41.67)	5 (20.83)	4 (16.67)		17 (70.83)
*Klebsiella pneumoniae*, *n* = 7; *n* (%)	7 (100.0)	7 (100.0)	7 (100.0)	7 (100.0)	6 (85.71)	2 (28.57)	6 (85.71)	6 (85.71)	0 (0.0)	0 (0.0)	0 (0.0)	1 (14.29)		2 (28.57)
*Pseudomonas aeruginosa*, *n* = 7; *n* (%)	7 (100.0)	7 (100.0)	7 (100.0)	7 (100.0)	7 (100.0)	4 (57.14)	7 (100.0)	4 (57.14)	7 (100.0)	0 (0.0)	5 (71.43)	0 (0.0)		7 (100.0)
*Salmonella* Typhi, *n* = 4; *n* (%)	4 (100.0)	2 (50.0)	3 (75.0)	4 (100.0)	2 (50.0)	1 (25.0)	3 (75.0)	3 (75.0)	2 (50.0)	1 (25.0)	0 (0.0)	1 (25.0)		3 (75.0)
*Shigella flexneri*, *n* = 3; *n* (%)	3 (100.0)	3 (100.0)	3 (100.0)	3 (100.0)	3 (100.0)	2 (66.67)	3 (100.0)	0 (0.0)	0 (0.0)	2 (66.67)	0 (0.0)	1 (33.33)		2 (66.67)
*Proteus vulgaris*, *n* = 1; *n* (%)	1 (100.0)	1 (100.0)	1 (100.0)	1 (100.0)	1 (100.0)	1 (100.0)	0 (0.0)	0 (0.0)	0 (0.0)	0 (0.0)	1 (100.0)	1 (100.0)		1 (100.0)
*Providencia alcalifaciens*, *n* = 1; *n* (%)	1 (100.0)	1 (100.0)	1 (100.0)	1 (100.0)	1 (100.0)	1 (100.0)	1 (100.0)	1 (100.0)	1 (100.0)	0 (0.0)	0 (0.0)	0 (0.0)		1 (100.0)
*Yersinia enterocolitica*, *n* = 1; *n* (%)	1 (100.0)	1 (100.0)	1 (100.0)	1 (100.0)	1 (100.0)	0 (0.00)	1 (100.0)	1 (100.0)	1 (100.0)	1 (100.0)	1 (100.0)	0 (0.0)		1 (100.0)

Abbreviations: AMP, ampicillin; AMX, amoxicillin; AZM, azithromycin; C, chloramphenicol; CFX, cefixime; CRX, cefuroxime; CTR, ceftriaxone; DOX, doxycycline; ERY, erythromycin; GN, gentamicin; PEN, penicillin; PIP, piperacillin; TET, tetracycline.

**Table 5:** Prevalence of multidrug‐resistant (MDR) Gram‐negative bacteria from COVID‐19 patients.

Name of isolates	Rates of MDR, *n* (%)
*Escherichia coli*	22/24 (91.67)
*Klebsiella pneumoniae*	6/7 (85.71)
*Pseudomonas aeruginosa*	7/7 (100.0)
*Salmonella* Typhi	2/4 (50.0)
*Shigella flexneri*	3/3 (100.0)
*Proteus vulgaris*	1/1 (100.0)
*Providencia alcalifaciens*	1/1 (100.0)
*Yersinia enterocolitica*	1/1 (100.0)
Total MDR Gram‐negative isolates	43/48 (89.58)
Resistant to 4 antibiotics	2/43 (4.65)
Resistant to 5 antibiotics	3/43 (6.98)
Resistant to 6 antibiotics	3/43 (6.98)
Resistant to 7 antibiotics	4/43 (9.30)
Resistant to 8 antibiotics	10/43 (23.26)
Resistant to 9 antibiotics	8/43 (18.60)
Resistant to 10 antibiotics	9/43 (20.93)
Resistant to 11 antibiotics	3/43 (6.97)
Resistant to 12 antibiotics	1/43 (2.33)

## 4 Discussion

The global impact of the emergence of SARS‐CoV‐2 has been devastating. Research and meta‐analyses have revealed that almost 75% of COVID‐19 patients consumed antibiotics, yet fewer than 10% also had fungal or bacterial coinfections. Due to improper usage of antibiotics, MDR bacteria have increased (Slimene et al. 2023; Su et al. 2022). This study revealed that 65.5% of patients (*n* = 19) consumed SM antibiotics during the COVID‐19 pandemic, similar to the study conducted in Iran (Faraji et al. 2022). This study also showed that 20.7% of patients (*n* = 6) consumed antibiotics during post‐COVID‐19 and 86.2% of patients (*n* = 25) consumed antibiotics during pre‐COVID‐19. Overall, this survey showed that a total of 86.2% patients (*n* = 25) practiced SM or doctor‐prescribed antibiotics in pre‐, during, and post‐COVID‐19 according to their diseases. Jirjees et al. described that the main attribute behind the high prevalence of SM with antibiotics can be due to cost‐saving measures, nervousness of COVID‐19 infection, quarantine conditions, rapid accessibility to antibiotics, lack of approved treatments, and the influence of social media, which contributed to panic among COVID‐19 patients (Jirjees et al. 2022). This study found that SM practices were significantly higher among university students with a mean age of (22.97 ± 2.542), which aligned highly with a cross‐sectional study, where 61.0% of participants had self‐medicated with antibiotics within the previous 6 months, and the highest rate of SM was obtained in between 22 and 25 years old group (Wahab et al. 2023). The maximum number of participants in this study was male (*n* = 26), and several studies suggest that males are more prone to SM of antibiotics than females (Ahmed et al. 2023). In this study, the high prevalence of SM of antibiotics may be driven by several factors, such as people's overconfidence due to their advanced education, which leads them to believe they have sufficient knowledge to diagnose and manage their problems. The desire for a fast cure from disease and unrestricted internet accessibility may also contribute to higher usage of SMA. Additionally, AMR may be influenced by the heavy hospital usage of antibiotics in COVID‐19 patients during the early stages of the pandemic, possibly because of similar illness patterns of viral diseases like the flu, common colds, and other respiratory illnesses (Baracaldo‐Santamaría et al. 2022; Faraji et al. 2022; Kimathi et al. 2022). Therefore, this study examined the impact of self‐medicated antibiotics and factors on the gut bacterial diversity of COVID‐19 patients.

Antibiotics have a profound impact on the homeostasis of the gut microbiota, disrupting its microbial community and affecting host health (Ribeiro et al. 2020). As a consequence, this study isolated 48 Gram‐negative opportunistic pathogenic bacteria from 29 specimens. Although the isolated bacterial strain aligns with the previous study conducted by Fernandes‐Pineda et al. (2024), the prevalence rate was quite different. The Prevalence of MDR Gram‐negative bacteria from COVID‐19 patients is represented in Table 5. The present study indicates that all the isolates showed resistance against most of the commonly used antibiotics, and surprisingly, 89.58% of the isolates (43/48) were found to be MDR. A study conducted in South Africa found that 50% of patients in tertiary hospitals had Gram‐negative MDR bacteria colonization upon admission (Asare Yeboah et al. 2023). Another study was conducted in Brazil, where 280 patients were hospitalized in intensive care units and the prevalence rate of Gram‐negative MDR bacteria was 54.4% (de Souza et al. 2023). It is possible that antibiotics were frequently used during COVID‐19 was selected for antibiotic‐susceptible tests, and the high percentages of SM among COVID‐19 patients contributed to the high prevalence of Gram‐negative MDR bacteria in this study. According to a survey, in North America, Europe, China, and Asia, the most often prescribed antibiotic groups were cephalosporins, β‐lactam/β‐lactamase inhibitors, fluoroquinolones, and macrolides (Akhtar et al. 2021), which showed similarity with this study. A recent study revealed that the emergence of resistant bacterial strains and the subsequent decline in microbiota diversity due to antibiotic therapy have become major challenges in the global battle against invasive infections and can even result in chronic side effects, including inflammatory bowel disease and asthma (Wuethrich et al. 2021).

In this study, COVID‐19 patients were divided into four groups based on their antibiotic consumption practices during the COVID‐19 period, pre‐COVID‐19, and post‐COVID‐19. The first group of patients was nonconsumers of antibiotics at pre‐, during, and post‐COVID‐19. The second group of patients consumed antibiotics only in pre‐COVID‐19 (doctor‐prescribed antibiotics). The third group of patients consumed antibiotics during and pre‐COVID‐19, with SM antibiotics during COVID‐19. On the other hand, the fourth group of patients consumed antibiotics pre‐, during (SM), and post‐COVID‐19. The fourth (Figure 4D) and third groups (Figure 4C) of patients who practiced SM antibiotics during COVID‐19 showed higher resistance patterns than the second group of patients (Figure 4B) who practiced doctor‐prescribed antibiotics during COVID‐19. Among the 13 antibiotics, *E. coli* showed resistance against 7–12 antibiotics (Figure 4D), 6–10 antibiotics (Figure 4C), and 4–5 antibiotics (Figure 4B). *K. pneumoniae* in the fourth group showed resistance against 9 antibiotics (Figure 4D), whereas the third and second groups showed resistance against 7–8 (Figure 4C) and 6 antibiotics (Figure 4B), respectively. These findings clearly state that the SM of antibiotics strongly impacts gut bacterial resistance. All *P. aeruginosa* isolated from the fourth and third groups showed resistance against 8–11 antibiotics (Figure 4C,D). The possible reasons behind the high resistance pattern of *P. aeruginosa* are due to its wide range of virulence genes and various antibiotic‐resistance pathways that contribute to becoming a potent and opportunistic pathogen, which confers a high risk to vulnerable groups of people like surgical patients, immunocompromised patients, Caucasians with cystic fibrosis, and so forth (Pelegrin et al. 2021). *Salmonella* spp., obtained from the third and second groups, exhibit resistance to 6–7 and 6 antibiotics, respectively. *P. vulgaris was* isolated from the fourth group, and *Y. enterocolitica* from the third group was found resistant to 8 (Figure 4D) and 11 (Figure 4C) antibiotics, respectively. Statistically, the Chi‐square test also showed a strong relation between antibiotic consumption timelines and antibiotic‐resistant patterns (*p* = 0.010; *p* < 0.05).

Some unusual results were observed in the first group of patients who did not consume antibiotics (Figure 4A). All isolates of *S. flexneri, Salmonella* Typhi, *P. alcalifaciens*, and one isolate of *E. coli* were resistant to 10 antibiotics. In contrast, *K. pneumoniae* and two isolates of *E. coli* showed resistance to 4 and 6 antibiotics, respectively, suggesting they are non‐MDR. Although these patients did not consume any antibiotics, their resistance patterns were higher, possibly because the antibiotic resistance profile depends not only on the SMA but also on other factors influencing the resistant profile of isolates. Report suggests that pets and wild animals can harbor AMR, and the use of antibiotics in livestock production to preserve health and productivity continues to spread infectious diseases and resistance genes through the environment and food (Miranda et al. 2020). This study only considered patients' antibiotic consumption history before, during, and after COVID‐19, which cannot provide insights into their entire antibiotic use over their lifetime. These patients may have acquired antibiotic resistance before these timeframes, which were not included in this study. Several studies suggest that other sources such as sanitation practices, poultry meats and eggs, street foods, and drinking water can also contribute to MDR characteristics. The gut microbiome acts as a reservoir for ARGs, which can lead to the development of bacterial resistance to antibiotics through horizontal gene transfer between commensals and opportunistic pathogens (Su et al. 2022). A study of six different water sources found antibiotic‐resistant bacterial isolates, including *Shigella* spp., *P. aeruginosa*, *E. coli*, *Klebsiella* spp., *Salmonella* Typhi, *P. vulgaris*, and *Vibrio cholerae*, with *E. coli* having MDR properties present in 49.48% of cases (Odonkor and Addo 2018). Another study examined 920 samples from chicken intestines and eggs, with 160 samples (17.4%) testing positive for *Salmonella* spp., which showed resistance to 2–5 antibiotics, and eight strains demonstrating resistance to six or more antimicrobial drugs (Hai et al. 2020).

The survey of this study found that 82.8% (*n* = 24) of patients drank locally supplied water, and 17.2% (*n* = 5) drank purified reverse osmosis water supplied by the university, along with 55.2% (*n* = 16) of participants involved in the processing of poultry meats or eggs before consumption. This survey revealed that 41.4% of participants (*n* = 12) consumed street food every day, 24.1% (*n* = 7) consumed it once a week, and 34.5% (*n* = 10) consumed it twice a week. The study revealed that only 37.9% (*n* = 11) maintained proper sanitation practices before eating any foods. It suggests that people can acquire AGRs from the above‐mentioned factors without practicing SMA. As a result, in this study, timelines (5–6 years), the patients who did not consume any antibiotics showed much more resistance than the expected ones. However, to control this worldwide epidemic, which impacts not only humans but also animals, plants, food, and the environment, a long‐term One Health approach is required to engage and unite everyone associated with this to pursue a common goal (Miranda et al. 2020).

Eight types of bacterial strains in this study were found to be resistant to amoxicillin, azithromycin, ampicillin, penicillin, erythromycin, and cefuroxime. All 48 isolates showed resistance to penicillin. In this study, the predominant bacterial isolates were *E. coli*, which exhibits resistance to all antibiotics. *P. aeruginosa*, *P. alcalifaciens*, *P. vulgaris*, and *Y. enterocolitica* showed resistance to the maximum antibiotics used. One of the important Enterobacteriaceae, *K*. *pneumoniae*, is thought to be an opportunistic pathogen that causes a wide range of illnesses, including septicemia, endocarditis, pneumonia, urinary tract infections, and cystitis. It exhibits an increased frequency of antibiotic resistance (Effah et al. 2020). However, this study revealed that *K. pneumoniae* was 100% resistant to amoxicillin, azithromycin, ampicillin, and penicillin, and 100% susceptible to ceftriaxone, gentamicin, and chloramphenicol. In 2016, research conducted in Pakistan discovered a widespread drug‐resistant *Salmonella* Typhi outbreak. The bacterial strains were resistant to ampicillin, trimethoprim/sulfamethoxazole, fluoroquinolones, chloramphenicol, and third‐generation cephalosporins. Recently, *Salmonella* Typhi was found to be resistant to ceftriaxone in Bangladesh, India, and the UK (Hooda et al. 2025). Ceftriaxone, a third‐generation antibiotic, was a key medication in treating typhoid for decades. The healthcare system is facing continuous pressure due to ceftriaxone resistance in *Salmonella* Typhi. In the present study, *Salmonella* spp. also showed 75% resistance to ampicillin and 50% to ceftriaxone, whereas it showed susceptibility to chloramphenicol. *S. flexneri* were 100% resistant to amoxicillin, azithromycin, ampicillin, penicillin, erythromycin, and cefixime, and 100% susceptible to piperacillin, ceftriaxone, and chloramphenicol.

This study investigated not only the impacts of antibiotic practices during three timelines with SM but also suggested other factors that could contribute to developing the antibiotic‐resistance pattern of gut bacteria among COVID‐19 patients. After all, being an educated person, they had information on antibiotics, but without a doctor's prescription, they consumed antibiotics by SM from different sources like pharmacies, family, friends, and so forth. Although this study showed a statistically significant correlation between antibiotic resistance patterns with consumption timelines (*p* = 0.01), consuming street foods (*p* = 0.004), maintaining sanitation (*p* = 0.011), and processing poultry meats and eggs (*p* = 0.026) before consuming and there was no significant correlation found with drinking water (*p* = 0.532), residents (*p* = 0.532), and pet animals (*p* = 0.117).

The results of this study suggested that, because of antibiotic misuse, the gut commensal bacterial species decrease, and opportunistic pathogenic bacterial species such as *K. pneumoniae*, *P. aeruginosa*, *S. flexneri*, and *Salmonella* spp. increased among people's gut, and most of them were MDR strains. MDR strains increase the treatment costs rather than susceptible ones, and also prolong hospital stays. So, the findings of this study will help raise awareness among the general people about the potential risk of AMR caused by MDR strains, which will be beneficial to the national economy by reducing treatment costs and prolonged hospital stays. Furthermore, this study will also increase awareness among policymakers and the infection prevention teams of hospitals, promoting the prevention of MDR strains from spreading to the environment and human health by implementing action plans to tackle the next pandemic potentially caused by AMR. The resistance patterns of this study can be stopped by maintaining SMA and a healthy lifestyle.

This study was conducted in a small region of Bangladesh, which cannot represent a nationwide scenario of antibiotic resistance patterns. Besides, the study had several limitations, including a small sample size and the lack of a molecular assay that could reveal the actual reason behind the mechanisms of antibiotic resistance. Further investigation with a larger sample volume focusing on molecular assays and associated factors should be conducted to create awareness among people about the misuse of antibiotics and help prevent the spread of AMR in hospitals and communities.

## 5 Conclusions

Since its emergence in Wuhan City, China in December 2019, SARS‐CoV‐2 has caused severe COVID‐19, resulting in a global epidemic within a few days. During the pandemic, SM with antibiotics frequently increased in LMICs, especially Bangladesh, due to fear of the virus, and inadequate antibiotic stewardship programs significantly affected gut bacteria. So, we evaluated the resistance patterns of gut bacteria in COVID‐19 patients and assessed the factors like SM of antibiotics, consuming street foods, having pet animals, being residents, source of drinking water, sanitation, and involvement of food processing, which were responsible for leading to antibiotic resistance. We obtained a higher prevalence of MDR opportunistic bacteria from the gut of COVID‐19 patients. We also noticed that self‐medicated COVID‐19 patients' gut bacteria showed more resistance to antibiotics than those who received doctor‐prescribed antibiotics. Our study will raise awareness among the general people about the potential risk of antibiotic resistance and MDR bacterial infections. A thorough understanding of the antibiotic resistance patterns in gut bacteria may aid medical personnel in more accurately prescribing antibiotics and possibly reducing hospital stays and medical costs. Moreover, our study will help the infection prevention team prevent the spread of AMR in the hospital and community settings.

## Author Contributions

**Rabeya Khanam:** conceptualization, methodology, investigation, writing – original draft, writing – review and editing, data curation, formal analysis. **Md. Yamun Hasan:** investigation, software, formal analysis, data curation, visualization, writing – review and editing, writing – original draft. **Abdul Malek:** investigation, writing – original draft, writing – review and editing, software, formal analysis, data curation. **Sazzad Hossain Sagor:** writing – original draft, writing – review and editing, investigation. **Chandan Sikder:** conceptualization, methodology, writing – review and editing. **Md. Sahabuddin:** conceptualization, methodology, formal analysis, writing – review and editing, visualization, resources, project administration, supervision, validation, funding acquisition, writing – original draft.

## Ethics Statement

The Research Ethics Committee of the Department of Biotechnology and Genetic Engineering (Registration No. BGE/REC/22/03) and the Research Center Ethics Review Committee (Registration No. BSMRSTU‐RC/ERC/22/027) of Gopalganj Science and Technology University, Goalaganj‐8100, Bangladesh, have approved this study. Before sample collection, participants were informed with information regarding the study's objectives, funding, and institution, and written informed consent was taken by physical interview.

## Conflicts of Interest

The authors declare no conflicts of interest.

## Supporting information


**Table S1:** Biochemical test results of isolated gram‐negative bacteria from COVID‐19 patients.

SPSS Test results.

Survey questions.

## Data Availability

Isolated bacterial 16S rRNA genome sequences were deposited in the NCBI GenBank database under accession numbers PP999312, PP999302, PP999159, PP999472, PP999444, PP999436, PQ000984, and PQ000246.
