# Supplementary material for: Examine the Impact of Self‐Medicated Antibiotics on Gut Bacterial Diversity From COVID‐19 Patients in Gopalganj, Bangladesh
Source: Microbiologyopen. 2025 Sep 30;14(5):e70063. doi: 10.1002/mbo3.70063 (PMC12481213; doi:10.1002/mbo3.70063)
Supplement: Supplementary file 1 — Table S1: Biochemical test results of isolated gram‐negative bacteria from COVID‐19 patients. [file MBO3-14-e70063-s003.docx]

**Table S1.** Biochemical test results of isolated gram-negative bacteria from COVID-19 patients

| **Sample No.** | **Identified Organism** | **Lactose Fermentation** | **Oxidase** | **Catalase** | **Methyl Red (MR)** | **Voges-Proskauer (VP)** | **Motility** | **Indole** | **Urease** |
| --- | --- | --- | --- | --- | --- | --- | --- | --- | --- |
| **S-2, S-5, S-7, S-8, S-9,**  **S-10, S-11, S-14, S-15, S-**  **16, S-17, S-18, S-19, S-20,**  **S-21, S-22, S-23, S-24, S-**  **25, S-26, S-28, S-30, S-31**  **and S-32** | *Escherichia coli* | (+ve) | (-ve) | (+ve) | (+ve) | (-ve) | (+ve) | (+ve) | (-ve) |
| **S-2, S-8, S-17, S-24, S-25, S-27 and S-32** | *Klebsiella pneumoniae* | (+ve) | (-ve) | (+ve) | (-ve) | (+ve) | (-ve) | (-ve) | (+ve) |
| **S-4, S-21, S-22, S-23, S-25, S-30 and S-32** | *Pseudomonas aeruginosa* | (-ve) | (+ve) | (+ve) | (-ve) | (-ve) | (+ve) | (-ve) | (-ve) |
| **S-8, S-12, S-13 and S-27** | *Salmonella spp.* | (-ve) | (-ve) | (+ve) | (+ve) | (-ve) | (+ve) | (-ve) | (-ve) |
| **S-8, S-9 and S-27** | *Shigella flexneri* | (-ve) | (-ve) | (+ve) | (+ve) | (-ve) | (-ve) | (-ve) | (-ve) |
| **S-1** | *Proteus vulgaris* | (-ve) | (-ve) | (+ve) | (+ve) | (-ve) | (+ve) | (+ve) | (+ve) |
| **S-24** | *Yersinia enterocolitica* | (+ve) | (-ve) | (+ve) | (+ve) | (-ve) | (-ve) | (+ve) | (+ve) |
| **S-16** | *Providencia alcalifaciens* | (-ve) | (-ve) | (+ve) | (+ve) | (-ve) | (+ve) | (+ve) | (-ve) |
