# Supplementary material for: Examine the Impact of Self‐Medicated Antibiotics on Gut Bacterial Diversity From COVID‐19 Patients in Gopalganj, Bangladesh
Source: Microbiologyopen. 2025 Sep 30;14(5):e70063. doi: 10.1002/mbo3.70063 (PMC12481213; doi:10.1002/mbo3.70063)
Supplement: Supplementary file 2 — SPSS Test results. [file MBO3-14-e70063-s002.docx]

| **Antibiotics Consumption Timelines * Antibiotic Resistant Level Crosstabulation** | | | | | | |
| --- | --- | --- | --- | --- | --- | --- |
|  |  |  | **Antibiotic-Resistant Level** | | | **Total** |
|  |  |  | **High** | **Low** | **Medium** |  |
| Antibiotics Consumption Timelines | 1timelines | Count | 0 | 2 | 5 | 7 |
|  |  | Expected Count | 2.9 | .6 | 3.5 | 7.0 |
|  | 2timelines | Count | 11 | 0 | 15 | 26 |
|  |  | Expected Count | 10.8 | 2.2 | 13.0 | 26.0 |
|  | 3timelines | Count | 5 | 0 | 3 | 8 |
|  |  | Expected Count | 3.3 | .7 | 4.0 | 8.0 |
|  | None | Count | 4 | 2 | 1 | 7 |
|  |  | Expected Count | 2.9 | .6 | 3.5 | 7.0 |
| Total | | Count | 20 | 4 | 24 | 48 |
|  |  | Expected Count | 20.0 | 4.0 | 24.0 | 48.0 |

| **Chi-Square Tests** | | | |
| --- | --- | --- | --- |
|  | **Value** | **df** | **Asymp. Sig. (2-sided)** |
| Pearson Chi- Square | 16.855^a^ | 6 | **.010** |
| Likelihood Ratio | 20.403 | 6 | **.002** |
| N of Valid Cases | 48 |  |  |
| a. 10 cells (83.3%) have expected count less than 5. The minimum expected count is .58. | | | |

| **Consumption of street food in week * Antibiotic Resistance Level Crosstabulation** | | | | | | |
| --- | --- | --- | --- | --- | --- | --- |
|  |  |  | **Antibiotic Resistance Level** | | | **Total** |
|  |  |  | **High** | **Low** | **Medium** |  |
| Consumption of street food in a week | Every day | Count | 14 | 2 | 4 | 20 |
|  |  | Expected Count | 8.3 | 1.7 | 10.0 | 20.0 |
|  | Once a week | Count | 1 | 0 | 9 | 10 |
|  |  | Expected Count | 4.2 | .8 | 5.0 | 10.0 |
|  | Twice a week | Count | 5 | 2 | 11 | 18 |
|  |  | Expected Count | 7.5 | 1.5 | 9.0 | 18.0 |
| Total | | Count | 20 | 4 | 24 | 48 |
|  |  | Expected Count | 20.0 | 4.0 | 24.0 | 48.0 |

| **Chi-Square Tests** | | | |
| --- | --- | --- | --- |
|  | **Value** | **df** | **Asymp. Sig. (2-sided)** |
| Pearson Chi- Square | 15.404^a^ | 4 | **.004** |
| Likelihood Ratio | 17.162 | 4 | **.002** |
| N of Valid Cases | 48 |  |  |
| a. 4 cells (44.4%) have expected count less than 5. The minimum expected count is .83. | | | |

| **Antibiotic resistance level * Having pet animals Crosstabulation** | | | | | |
| --- | --- | --- | --- | --- | --- |
|  |  |  | **Having pet animals** | | **Total** |
|  |  |  | **No** | **Yes** |  |
| Antibiotic resistance level | High | Count | 19 | 1 | 20 |
|  |  | Expected Count | 16.2 | 3.8 | 20.0 |
|  | Low | Count | 3 | 1 | 4 |
|  |  | Expected Count | 3.2 | .8 | 4.0 |
|  | Mediu m | Count | 17 | 7 | 24 |
|  |  | Expected Count | 19.5 | 4.5 | 24.0 |
| Total | | Count | 39 | 9 | 48 |
|  |  | Expected Count | 39.0 | 9.0 | 48.0 |

| **Chi-Square Tests** | | | |
| --- | --- | --- | --- |
|  | **Value** | **df** | **Asymp. Sig. (2-sided)** |
| Pearson Chi- Square | 4.294^a^ | 2 | **.117** |
| Likelihood Ratio | 4.914 | 2 | **.086** |
| N of Valid Cases | 48 |  |  |
| a. 4 cells (66.7%) have expected count less than 5. The minimum expected count is .75. | | | |

| **Drinking water * Antibiotic Resistance Level Crosstabulation** | | | | | | |
| --- | --- | --- | --- | --- | --- | --- |
|  |  |  | **Antibiotic Resistance Level** | | | **Total** |
|  |  |  | **High** | **Low** | **Mediu m** |  |
| Drinking water | Locally supplied water | Count | 15 | 4 | 19 | 38 |
|  |  | Expected Count | 15.8 | 3.2 | 19.0 | 38.0 |
|  | University supplied water | Count | 5 | 0 | 5 | 10 |
|  |  | Expected Count | 4.2 | .8 | 5.0 | 10.0 |
| Total | | Count | 20 | 4 | 24 | 48 |
|  |  | Expected Count | 20.0 | 4.0 | 24.0 | 48.0 |

| **Chi-Square Tests** | | | |
| --- | --- | --- | --- |
|  | **Value** | **df** | **Asymp. Sig. (2-sided)** |
| Pearson Chi- Square | 1.263^a^ | 2 | **.532** |
| Likelihood Ratio | 2.070 | 2 | **.355** |
| N of Valid Cases | 48 |  |  |
| a. 3 cells (50.0%) have expected count less than 5. The minimum expected count is .83. | | | |

| **Maintain sanitation * Antibiotic Resistance Level Crosstabulation** | | | | | | |
| --- | --- | --- | --- | --- | --- | --- |
|  |  |  | **Antibiotic Resistance Level** | | | **Total** |
|  |  |  | **High** | **Low** | **Mediu m** |  |
| Maintain sanitation | No | Count | 17 | 3 | 10 | 30 |
|  |  | Expected Count | 12.5 | 2.5 | 15.0 | 30.0 |
|  | Yes | Count | 3 | 1 | 14 | 18 |
|  |  | Expected Count | 7.5 | 1.5 | 9.0 | 18.0 |
| Total | | Count | 20 | 4 | 24 | 48 |
|  |  | Expected Count | 20.0 | 4.0 | 24.0 | 48.0 |

| **Chi-Square Tests** | | | |
| --- | --- | --- | --- |
|  | **Value** | **df** | **Asymp. Sig. (2-sided)** |
| Pearson Chi- Square | 9.031^a^ | 2 | **.011** |
| Likelihood Ratio | 9.502 | 2 | **.009** |
| N of Valid Cases | 48 |  |  |
| a. 2 cells (33.3%) have expected count less than 5. The minimum expected count is 1.50. | | | |

| **Processing poultry and egg * Antibiotic Resistance Level Crosstabulation** | | | | | | |
| --- | --- | --- | --- | --- | --- | --- |
|  |  |  | Antibiotic Resistance Level | | | Total |
|  |  |  | High | Low | Mediu m |  |
| Processing poultry and egg | No | Count | 6 | 4 | 13 | 23 |
|  |  | Expected Count | 9.6 | 1.9 | 11.5 | 23.0 |
|  | Yes | Count | 14 | 0 | 11 | 25 |
|  |  | Expected Count | 10.4 | 2.1 | 12.5 | 25.0 |
| Total | | Count | 20 | 4 | 24 | 48 |
|  |  | Expected Count | 20.0 | 4.0 | 24.0 | 48.0 |

| **Chi-Square Tests** | | | |
| --- | --- | --- | --- |
|  | Value | df | Asymp. Sig. (2-sided) |
| Pearson Chi-Square | 7.296^a^ | 2 | **.026** |
| Likelihood Ratio | 8.920 | 2 | **.012** |
| N of Valid Cases | 48 |  |  |
| a. 2 cells (33.3%) have expected count less than 5. The minimum expected count is 1.92. | | | |

| **Residents * Antibiotic Resistance Level Crosstabulation** | | | | | | |
| --- | --- | --- | --- | --- | --- | --- |
|  |  |  | **Antibiotic Resistance Level** | | | **Total** |
|  |  |  | **High** | **Low** | **Medium** |  |
| Drinking water | Local Mess | Count | 15 | 4 | 19 | 38 |
|  |  | Expected Count | 15.8 | 3.2 | 19.0 | 38.0 |
|  | University Hall | Count | 5 | 0 | 5 | 10 |
|  |  | Expected Count | 4.2 | .8 | 5.0 | 10.0 |
| Total | | Count | 20 | 4 | 24 | 48 |
|  |  | Expected Count | 20.0 | 4.0 | 24.0 | 48.0 |

| **Chi-Square Tests** | | | |
| --- | --- | --- | --- |
|  | **Value** | **df** | **Asymp. Sig. (2-sided)** |
| Pearson Chi- Square | 1.263^a^ | 2 | **.532** |
| Likelihood Ratio | 2.070 | 2 | **.355** |
| N of Valid Cases | 48 |  |  |
| a. 3 cells (50.0%) have expected count less than 5. The minimum expected count is .83. | | | |
