# Supplementary material for: Examine the Impact of Self‐Medicated Antibiotics on Gut Bacterial Diversity From COVID‐19 Patients in Gopalganj, Bangladesh
Source: Microbiologyopen. 2025 Sep 30;14(5):e70063. doi: 10.1002/mbo3.70063 (PMC12481213; doi:10.1002/mbo3.70063)
Supplement: Supplementary file 3 — Survey questions. [file MBO3-14-e70063-s001.pdf]

Department of Biotechnology and Genetic Engineering
Bangabandhu Sheikh Mujibur Rahman Science and Technology University,
Gopalganj-8100, Bangladesh.

Name of the participants:

Sex: Phone Number:

Address:

Q1. Have you been affected by COVID-19?

A. Yes
B. No

Q2. Have you been diagnosed with coronavirus symptoms?

A. Yes
B. No

If your answer is **Yes** on (Q2)	If your answer is **No** on (Q2)
2.1 Have you been admitted to the hospital?<br>A. Yes<br>B. No	2.1: Did you have symptoms of coronavirus?<br>A. Yes<br>B. No
2.2 Have you been prescribed antibiotics for COVID-19?<br>A. Yes<br>B. No	2.2: Have you taken any Antibiotics?<br>A. Yes<br>B. No
2.3 Have you completed your antibiotic dose?<br>A. Yes<br>B. No	2.3: Was it self-medicated or doctor-prescribed?<br>A. Yes<br>B. No
2.4 How long has your treatment continued? (7 Days / 14 Days / more)<br><br>Ans:	2.4: How long have you taken antibiotics? (7 Days / 14 Days / more)<br><br>Ans:
2.5 Which type of antibiotics have you taken?<br><br>Ans:	2.5: Which type of antibiotics have you taken?<br><br>Ans:

Q3. Have you taken any antibiotics within 6 months?

A. Yes
B. No

Q3.1 If YES, which type of Antibiotics have you taken?

Ans:

Q3.2: Is this Self-medicated or doctor-prescribed?

Ans:

Q4. How many times do you eat street foods?

A. Once a week
B. Twice a week
C. Every day

Q5. Do you have any pet animals?

A. Yes
B. No

Q6. Where do you live?

A. Local Mess
B. University Hall

Q7. Source of your drinking water?

A. Locally supplied water
B. University supplied reverse osmosis water

Q8. Do you properly maintain sanitation before eating food?

A. Yes
B. No

Q9. Do you process poultry meats or eggs before consumption?

A. Yes
B. No

Q10. Pre-covid antibiotic consumption frequency rate (last 2-3 years before 2021)?

Ans:
